# Flexible nano-liposomes-based transdermal hydrogel for targeted delivery of dexamethasone for rheumatoid arthritis therapy

**DOI:** 10.1080/10717544.2022.2096718

**Published:** 2022-07-10

**Authors:** Yi-Pu Zhao, Jiang-Fan Han, Fei-Yue Zhang, Tian-Tian Liao, Ren Na, Xiao-Feng Yuan, Guang-bin He, Weiliang Ye

**Affiliations:** a Department of Pharmaceutics, School of Pharmacy, Fourth Military Medical University, Xi’an, China; b Lab for Bone Metabolism, Key Lab for Space Biosciences and Biotechnology, School of Life Sciences, Northwestern Polytechnical University, Xi’an, China; c Department of Epidemiology and Health Statistics, Faculty of Military Preventive Medicine, Fourth Military Medical University, Xi’an, China; d Department of Ultrasound Diagnosis, Xijing Hospital, Fourth Military Medical University, Xi’an, China

**Keywords:** Transdermal drug delivery system, dextran sulfate, flexible liposome, rheumatoid arthritis

## Abstract

Rheumatoid arthritis (RA) is an inflammatory immune-mediated disease that can lead to synovitis, cartilage destruction, and even joint damage. Dexamethasone (DEX) is a commonly used agent for RA therapy on inflammation manage. However, the traditional administering DEX is hampered by low efficiency and obvious adverse effects. Therefore, in order to efficiently deliver DEX to RA inflamed joints and overcome existing deficiencies, we developed transdermal formation dextran sulfate (DS) modified DEX-loaded flexible liposome hydrogel (DS-FLs/DEX hydrogel), validated their transdermal efficiency, evaluated its ability to target activated macrophages, and its anti-inflammatory effect. The DS-FLs/DEX exhibited excellent biocompatibility, sustainable drug release, and high uptake by lipopolysaccharide (LPS)-activated macrophages. Furthermore, the DS-FLs/DEX hydrogel showed desired skin permeation as compared with regular liposome hydrogel (DS-RLs/DEX hydrogel) due to its good deformability. In vivo, when used the AIA rats as RA model, the DS-FLs/DEX hydrogel can effectively penetrate and accumulate in inflamed joints, significantly improve joint swelling in RA rats, and reduce the destructive effect of RA on bone. Importantly, the expression of inflammatory cytokines in joints was inhibited and the system toxicity did not activate under DS-FLs/DEX hydrogel treatment. Overall, these data revealed that the dextran sulfate (DS) modified DEX-loaded flexible liposome hydrogel (DS-FLs/DEX hydrogel) can prove to be an excellent drug delivery vehicle against RA.

## 1. Introduction

Rheumatoid arthritis (RA) is a systemic autoimmune disease characterized by long-term synovial dysplasia, joint inflammation, and erosion of periarticular bone (Martinsson et al., 2021). RA is one of the chief reasons accounting for the labor force loss of middle-aged people and thus affects patients’ quality of life and leads heavy burden for economic (Hu et al., 2018; Safiri et al., 2019). The RA pathophysiology is allied with an imbalance of the immune system, which leading to the activation of macrophages, generation of inflammatory microenvironment and damage of cells and tissues (Shim et al., 2018). Currently, no treatment has been demonstrated to reverse the progression of joint structural damage in RA patients. Effective joint pain control and inflammation management are the primary goals of cure disease (Helmick et al., 2008). Osteoarthritis Research Society International (OARSI) guidelines recommend intra-articular (IA) administration of glucocorticoids (GC) as an appropriate treatment for RA (zeng, 2018). However, the short-term anti-inflammatory activity of GC formulation and obvious toxic side effects seriously hinder its therapeutic effect on RA. Therefore, there requires an unmet clinical need for effective and safe GC therapy that can provide sustained pain relief and joint inflammation suppression for the clinical treatment of RA.

Transdermal drug delivery system (TDDS) is a noninvasive method that can avoid the liver first pass effect and prevent gastrointestinal irritation (Alexander et al., 2012). TDDS formulation is a prospective approach for achieving effective therapeutic on the RA long-term treatment, which maintain blood concentration for sustained release effect, prevent gastro intestinal tract side effect and reduce dosing frequency (Amjadi et al., 2018). However, due to the major obstacles to transdermal delivery of stratum corneum, only the drugs molecular weight of lower than 500 Da can easily permeate across the corium layer (Shen et al., 2021). Thus, to realize effectively improvement of GC transdermal permeation is necessary before the application of transdermal GC formulations.

Encapsulation of GC into flexible liposomes (FLs) is a promising way to resolve the above problem. FLs are a new generation of liposomes, which contain edge activators like Tween 80, cholate, deoxycholate and so on (Ascenso et al., 2015). The presence of edge activators could largely increase lipid layers elasticity, thereby destabilized lipid layers show more effective penetration into deep skin layers than conventional liposomes with rigid membranes (Badran et al., 2012). On the other hand, high membrane hydrophilicity protects FLs from fusion and aggregation under osmotic stress that makes it difficult with the traditional liposomes (Deak, 2014). Following the osmotic concentration gradient, FLs penetrate deeper epidermis layers through lipid lamellar areas of stratum corneum, but traditional liposomes fuse with the skin lipids, dehydrate and retention near the skin surface (Amnuaikit et al., 2018). Based on these advantages, FLs formulations have been developed for transdermal administration of multiple bioactive compounds, including nucleic acids, proteins (Abdulbaqi et al., 2016), and small molecule drugs (Nayak & Tippavajhala, 2021).

**Scheme 1.**
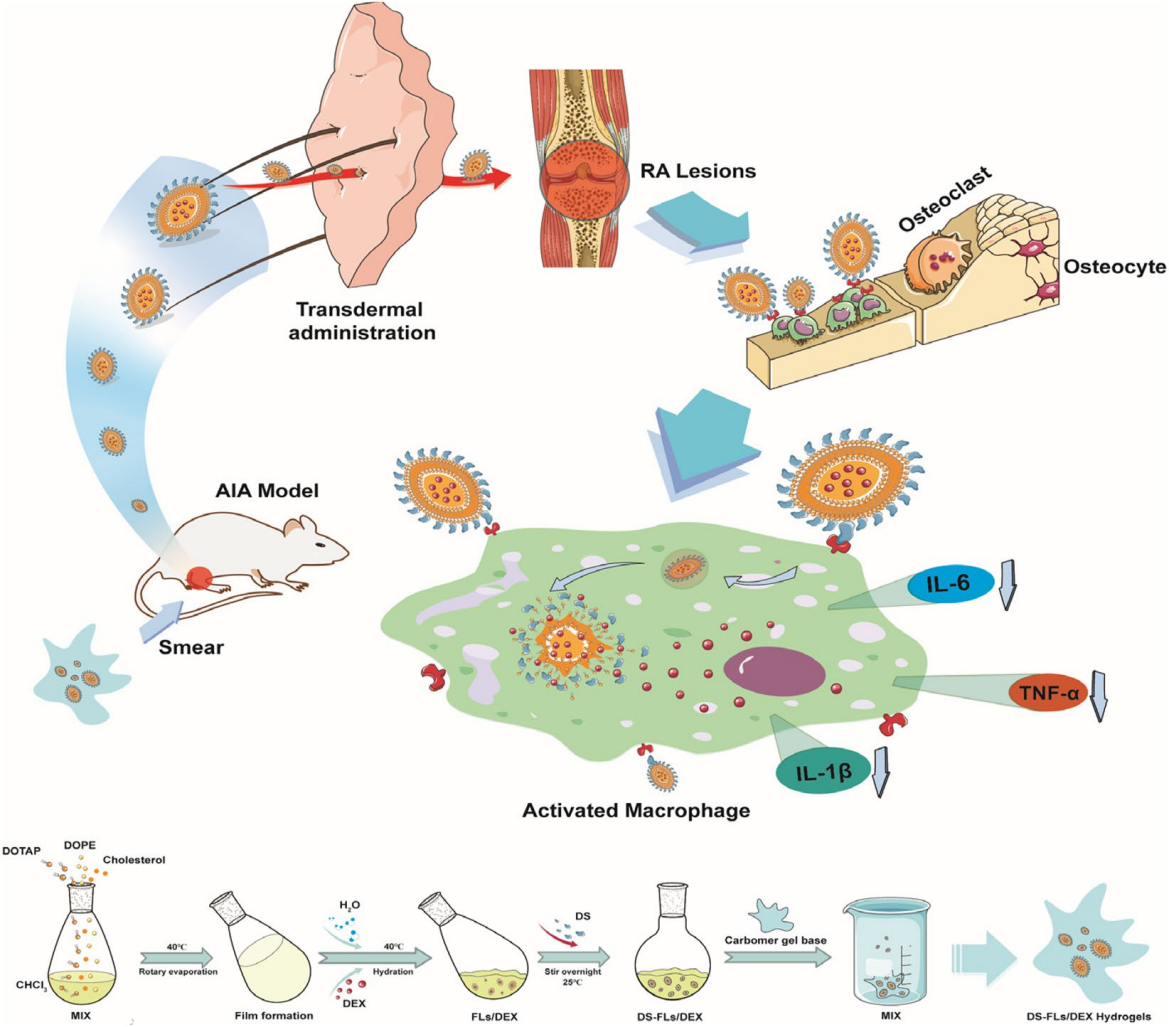
Schematic illustration of preparation and therapeutic mechanism against RA of DS-FLs/DEX hydrogels.

Herein, we design a dexamethasone (DEX)-loaded flexible liposomes (DS-FLs/DEX), DEX is widely used as an anti-inflammatory and immunosuppressive GC in RA treatment, as well as, polyanion compound dextran sulfate (DS) is the ligands of class A scavenger receptors (SR-A) and has good biocompatibility (Yang et al., 2017). Among many researches, DS was used to target SR-A overexpressed macrophages and the highly targeting efficiency was confirmed (You et al., 2014; Zhao et al., 2017). Subsequently, the DS-FLs/DEX was incorporated within a carbomer-based gel (DS-FLs/DEX hydrogel) for topical/transdermal treatment of RA. The bio-adhesive and nonirritant properties of carbomer make it has the potential to form a smooth, elegant and stable gel (Alami-Milani et al., 2018). There is no doubt that DS-FLs/DEX hydrogel provides a convenient method for drug application, and widely increases the drug retention time in the skin, which is conducive to the long-term therapy. Due to its extremely deformability, DS-FLs/DEX can easily penetrate into the deep skin layer, realizing a high accumulation of drug in diseased joint. The accumulated DS-FLs/DEX in the inflamed joint can specifically target to the SR-A on activated macrophages by DS modification, thereby amplifying the anti-inflammatory ability and reducing side effects of DEX, as shown in Scheme 1. Thus, we believe that DS-FLs/DEX hydrogel is a promising novel TDDS preparation for effective RA therapy.

## 2. Materials and methods

### 2.1. Materials

Cholesterol, carbomer 904, 1,2-dioleoyl-3-trimethylammonium-propane chloride salt (DOTAP), Dextran Sulfate (DS, MW5000 Da), deoxysodium cholate (NaDC), 1,2-dioleoyl-sn-glycero-3-phosphoethanolamine (DOPE), coumarin-6 (C-6), and dexamethasone (DEX) were purchased from J&K CHEMICA (Beijing, China). 4′,6-diamidino-2-phenylindole, Dulbecco’s Modified Eagle Medium (DMEM), Fetal bovine serum (FBS), 1,1-dioctadecyl-3,3,3,3-tetramethylindotricarbocyaineiodide (DIR), and lipopolysaccharides (LPS) reagent were from Invitrogen Technologies Company (Carlsbad, USA).

### 2.2. Cell culture and animals

RAW264.7 cells were a gift from the School of Life Sciences, Northwestern Polytechnical University (Xi’an, China) and cultured in DMEM in cell incubator (37 °C, 5% CO_2_). Male SD rats (weight = 180–200 g) were bought from the Air Force Medical University Animal laboratory center and adaptively fed one week before experiments. All procedures are in accordance with the guidelines for the care and use of experimental animals issued by the Air Force Medical University and approved by the Animal Ethics Committee (Xi’an, China).

### 2.3. Preparation of DS-FLs/DEX

DS-FLs/DEX were fabricated by a thin-film hydration method. Briefly, moderate DOTAP, DOPE, CHOL, and DEX were dissolved in a mixture solution of methanol and chloroform (6 mL, v/v = 3:1). Then, the organic solvents were removed by evaporation for 1 h in 40 °C conditions. Then the thin-film were vacuum drying for 1 h, and stored in 4 °C overnight. The obtained thin-film was then hydrated with 5 mL PBS phosphate solution with moderate edge activators and DS solution for 2 h to get the DS-FLs/DEX. In order to obtain uniformly distributed liposomes, the above mixture was extruded through 0.22 μm polycarbonate membranes for 5 times. Finally, the unloaded DEX was removed by ultra-filtration (×5000 rpm, 20 min) from DS-FLs/DEX. In order to optimal the DS-FLs/DEX formulation, the edge activators species, mass ratio of DOTAP:edge activators, mass ratio of DOTAP:CHOL and DEX concentration were investigated under single factor way (Alami-Milani et al., 2018). The DS-RLs/DEX without any edge activators was prepared for the comparator.

### 2.4. Characterization of DS-FLs/DEX

The size, PDI, and ξ-potential of DS-FLs/DEX were determined by dynamic light scattering (DLS, Nano ZS/ZEN3600, Malvern Panalytical, UK). The morphology of flexible liposome was measured by transmission electron microscopy (TEM, TECNAI Spirit G2, Thermo Fisher, USA). The encapsulation efficiency (EE) was carried out by HPLC. The stability of DS-FLs/DEX (4 mg/mL) in PBS was evaluated by monitoring the change of size and PDI value for 10 days at 37 °C via Beckman Coulter Particle Analyzer. The deformability index of DS-FLs/DEX was measured by an extrusion method (Lin et al., 2019). Briefly, the DS-FLs/DEX were extruded through a Whatman™ 50 nm Nuclepore™ Polycarbonate Track-Etched Membrane Filter (Whatman, UK) by applying pressure of 0.5 MPa for 15 min.

### 2.5. Biocompatibility of DS-FLs/DEX

The MTT assay was executed to evaluate the safety of the DS-FLs/DEX. The HUVEC cells were seeded in 96-well plates and incubated for 24 h. Then the cells were treated with various formulations for 24 h to measure the cellular activity. In the end, the absorbance in the well was measured by using a Microplate Reader (Bio-Rad Laboratories, Hercules, CA).

Hemolytic rate is a key criterion for the biocompatibility of the material (Cao et al., 2017; Qindeel et al., 2020). 100 μL diluted rat blood was added into 1 mL formulation solution, and the mixture were incubated for 60 min at 37 °C. After a series of processing, the OD value of the supernatant was measured by using UV-spectrophotometer. The physiological saline and 0.1% SDS were used as negative and positive control, respectively.

### 2.6. In vitro drug release

The DEX release profiles in DS-FLs/DEX were measured by a dialysis method. Briefly, 10 mg DS-FLs/DEX was placed in a dialysis bag (MW = 1000 kDa) and dialyzed under a constant stirring at 37 °C against PBS at different pH values (pH = 7.4 and 6.5). The dialysis bag outer medium was withdrawn at a predetermined time, and afterwards the same volume of fresh medium was added. The concentration of DEX was determined by HPLC.

### 2.7. Cellular uptake

The flexible liposome labeled with the DIR were used to observe the cellular uptake behaviors by using the fluorescence microscope (Nikon Corporation, Tokyo, Japan). RAW264.7 cells were seeded in a 12-well plate 1 × 10^5^ cells per well and activated by LPS. After 12 h of incubation, the cells were treated with DS-RLs/DEX and DS-FLs/DEX (DEX = 5 μg/mL) respectively, which were then continued to incubate for a predetermined time (0.5 h, 3 h). Then, the cells were stained with a certain concentration of DAPI for 10 min. Finally, after a series of operations, the fluorescence uptake of cells was observed.

### 2.8. In vitro anti-inflammatory activities

RAW264.7 cells were plated in 6-plates 2 × 10^6^ cells per well and activated by LPS at a certain concentration. After 12 h incubation, the cells were treated with DS-RLs/DEX and DS-FLs/DEX (DEX = 5 μg/mL), respectively. After another 6 h incubation, the levels of TNF-α, IL-1β, and IL-6 were determined by the ELISA kit.

### 2.9. Preparation of DS-FLs/DEX hydrogel

In order to facilitate the usage of DS-FLs/DEX on the skin, the DS-FLs/DEX loaded hydrogel was prepared as the previous reported method (Qindeel et al., 2020). In brief, 400 mg of Carbopol 934 was dissolved in 10 mL water and left to completely swell for overnight. Then, DS-FLs/DEX in water (1 mL) was incorporated into the Carbopol gel solution. The pH value was adjusted by triethanolamine (0.8 wt%). In the end, the homogeneous dispersion DS-FLs/DEX hydrogel was obtained. The DS-RLs/DEX hydrogel was prepared for the comparator.

### 2.10. Characterization of DS-FLs/DEX hydrogel

The color, appearance, and homogeneity of DS-FLs/DEX hydrogel were evaluated. The properties of the DS-FLs/DEX in the hydrogel (i.e. particle size, PDI, ζpotential, and encapsulation efficiency) were determined by dynamic light scattering (DLS, Nano ZS/ZEN3600, Malvern Panalytical, UK). The pH value of DS-FLs/DEX hydrogel was measured by a pH meter (OAKTON PH 700 Benchtop, pH meter). The drug loading, viscosity, and spreadability of DS-FLs/DEX hydrogel were also evaluated as the previously reported method (Mir et al., 2019; Qindeel et al., 2019).

### 2.11. In vitro skin permeation and retention

In vitro permeation studies were performed by using the Franz diffusion cell method. Skin samples were taken from the back of SD rats, and the cuticle of the skin was transdermally administered with DIR-DS-RLs/DEX hydrogel and DIR-DS-FLs/DEX hydrogel. At specified time intervals, 1 mL of the diffusion medium was removed from the donor chambers and the equal volume of fresh PBS was replenished. The content of DEX in the diffusion medium was determined by HPLC. After the study was completed, all skin surfaces were carefully washed with distilled water to remove the hydrogel residue on the skin surface, and the DEX in the skin was extracted with a tissue homogenizer, and the content of retained DEX in the skin was detected by HPLC.

### 2.12. In vivo skin permeation and accumulation study

In order to test the permeation properties of DS-RLs/DEX hydrogel by fluorescence intensity, the DIR-DS-RLs/C6 hydrogel and DIR-DS-FLs/C6 hydrogel were prepared and transdermally administered on the rats. After treatments, the rats were sacrificed to obtain the skins, and all skin surfaces are carefully cleaned with distilled water to remove hydrogel residue. They were then sliced and observed by using the fluorescence microscope (Nikon Corporation, Tokyo, Japan).

The DIR-labeled liposome hydrogel was prepared for further in vivo skin accumulation study. SD rats were trans-dermally administered on the back skin after hair removal with DIR-DS-RLs/DEX hydrogel and DIR-DS-FLs/DEX hydrogel at a dosage of 0.2 μg DIR/kg per rat (*n* = 3). To further investigate the accumulation properties of DS-RLs/DEX hydrogel at the RA joint site, the AIA rats were also trans-dermally administered DIR-DS-RLs/DEX hydrogel in joints (*n* = 3). After four hours administered, the rats were observed by using an IVIS® Spectrum system (Lumina, PerkinElmer, CA). The intensity of DIR fluorescence was also calculated by using the Living Imaging software (PerkinElmer, CA).

### 2.13. In vivo pharmacokinetics study

The AIA rats were randomly divided into four groups to investigate the pharmacokinetics of the different DEX formulations. The formulations were then administered orally or trans-dermally with the dose of 1 mg/kg. At predetermined time points after the administration, 0.4 mL of blood samples was obtained by glass capillary from the orbit and removed into the centrifugal tube containing heparin sodium. After centrifugation at 3,000 rpm for 10 min, the serum was separated and the amount of DEX was detected by HPLC.

### 2.14. Therapeutic efficacy

The rats were randomly divided into four groups (*n* = 5). Group 1 normal control, group 2 negative control (AIA rats), group 3 AIA rats treated with DS-RLs/DEX hydrogel and group 4 AIA rats treated with DS-FLs/DEX hydrogel (DEX equivalent to 1 mg/kg). All groups administered every 2 days, the body weight, paw thickness, and clinical scoring were recorded every 2 days. After the treatment, X-ray analysis was performed on the right thigh of the rats, and expression levels of TNF-α, IL-6, and IL-1β in joint tissue were detected by the ELISA kit.

### 2.15. Histological analysis

After treatments, the rats were sacrificed. Then the skin of the ankle joint, ankle joints, liver, and kidney were stained with H&E and observed by a light microscope (Model IX71 Olympus, Tokyo, Japan).

### 2.16. Safety evaluation

To evaluate the safety of each formulation, The levels of the blood glucose, alanine transaminase (ALT), aspartate aminotransferase (AST), blood urea nitrogen (BUN), and creatinine (Crea) in serum were analyzed by the biochemical auto-analyzer (AU5800, Beckman, Inc.) to evaluate the safety of formulations.

### 2.17. Statistical analysis

SPSS13.0 software was used to analyze the datum. Results were expressed as mean ± SD. For all experimentation, error bar indicates SD. Significance was determined using *t* test or one-way analysis of variance (ANOVA). A *p* value <0.05 was considered statistically significant (GraphPad).

## 3. Results and discussion

### 3.1. Formulation screening of DS-FLs/DEX

**Figure 1.**
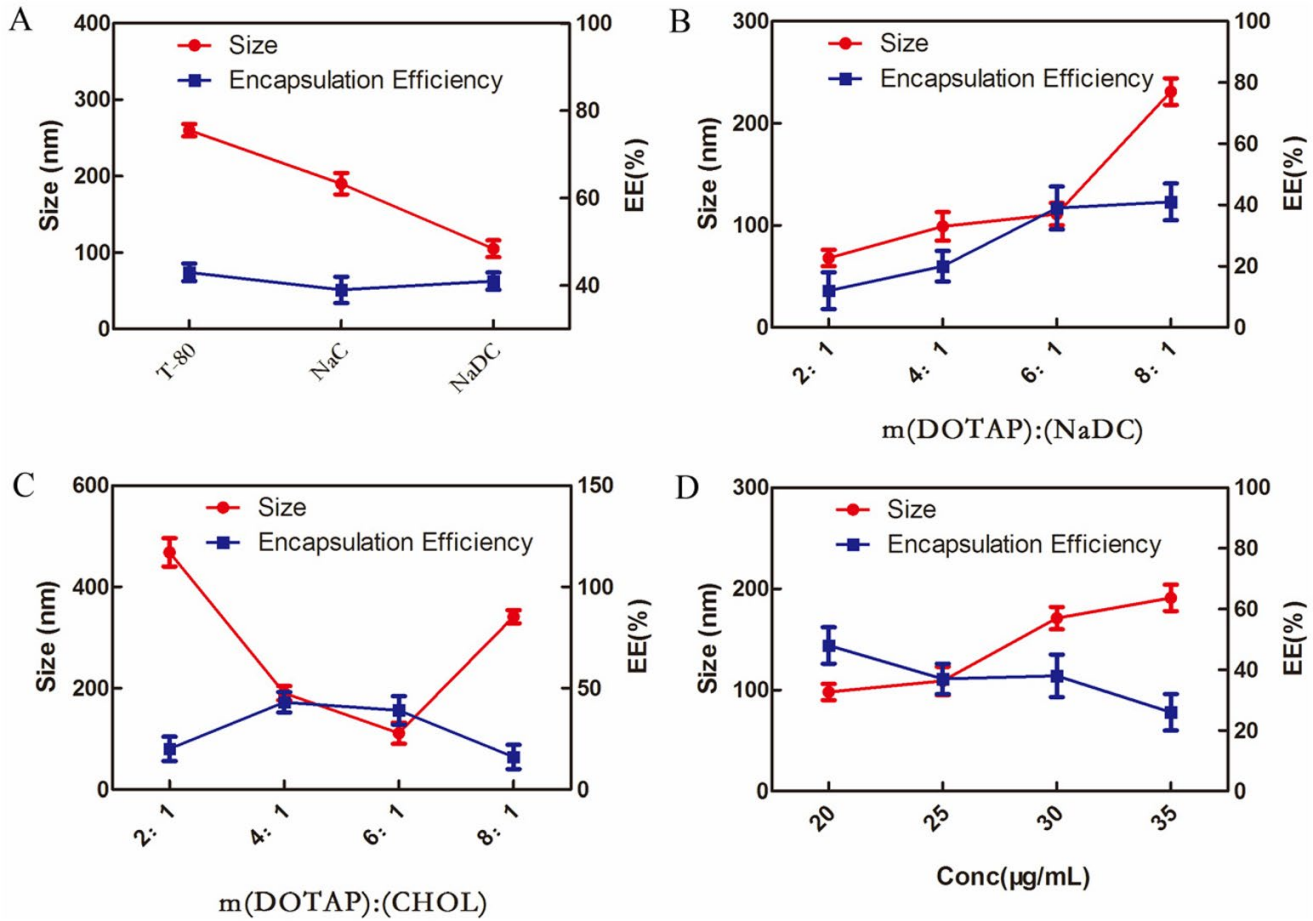
Size and encapsulation efficiency (EE) analysis of flexible liposome at (A) different edge activators, (B) different w:w ratios ranging from 2:1 to 8:1 for DOTAP: NaDC, (C) different w:w ratios ranging from 2:1 to 8:1 for DOTAP: CHOL, (D) different concentration from 20 to 35 for DEX.

The prescription screening criteria for liposome preparation are the particle size and EE, since both of the parameters have a significant impact on drugs permeation and accumulation through the skin. According to the standard of smaller size and higher EE, the formulations were optimized in four major aspects. As shown in Figure 1, NaDC was chosen as an edge activator. Meanwhile, m(DOTAP):m(NaDC)=6:1, m (DOTAP):m(CHOL)=6:1 and C(DEX)=20 μg/mL were selected for subsequent research. In the next step, two liposomes were prepared according to the above screening conditions.

### 3.2. Characterization of DS-FLs/DEX

**Figure 2.**
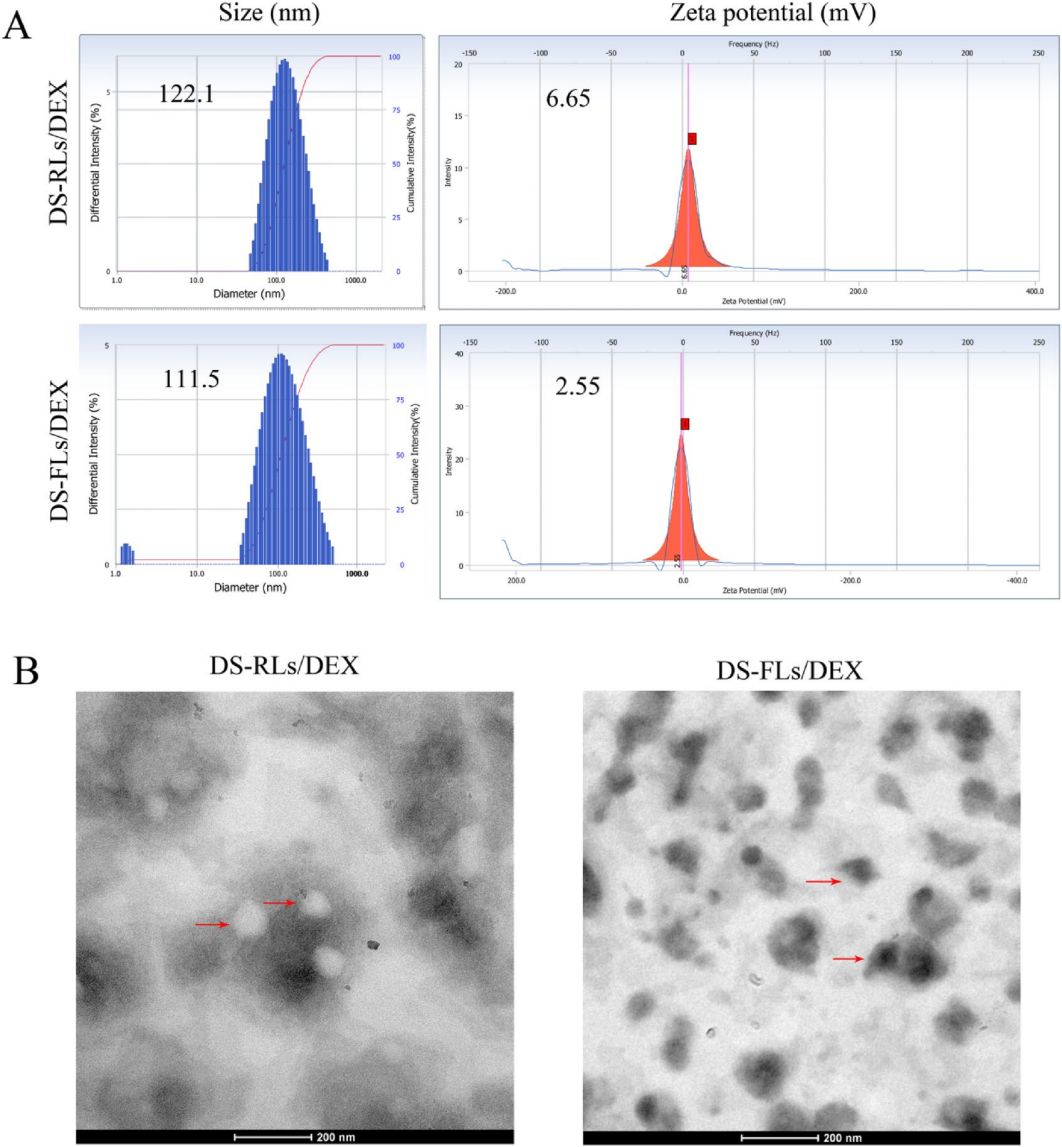
Particle size distribution and zeta potential (A) of DS-RLs/DEX and DS-FLs/DEX, (B) TEM images of DS-RLs/DEX and DS-FLs/DEX, and the lipsomes were marked with red arrows.

The results of the mean size and zeta potential of DS-RLs/DEX and DS-FLs/DEX were listed in Figure 2(A), and shown that the mean particle size was 122.1 nm and 112.5 nm, respectively. Besides, the zeta potential of DS-RLs/DEX and DS-FLs/DEX were 6.65 and 2.55 mV, respectively. TEM examinations showed that spherical morphology of DS-RLs/DEX, fusiform morphology of DS-FLs/DEX, and they had similar particle sizes as the DLS results (Figure 2(B)). Studies have shown that compared with spherical nanoparticles, fusiform nanoparticles are more conducive to the penetration of skin tissue, thereby enhancing the efficiency of drug transdermal delivery. Other characterization of liposomes is shown in Table 1, DS-RLs/DEX and DS-FLs/DEX had a polydispersity index (PDI) value of 0.215 and 0.196, encapsulation efficiency of 41% and 43%, and drug loading of 9.5% and 10.1%.

**Table 1.** The characterization of DEX-loaded lipsomes.

DEX loaded lipsomes	Size (nm)	Zeta (mV)	PDI	DL (%)	EE (%)
DS-RLs/DEX	122 ± 18	+6.65 ± 1.5	0.215 ± 0.09	9.5 ± 1.9	41 ± 2.1
DS-FLs/DEX	112 ± 13	+2.55 ± 1.3	0.196 ± 0.6	10.1 ± 1.6	43 ± 3.4

**Figure 3.**
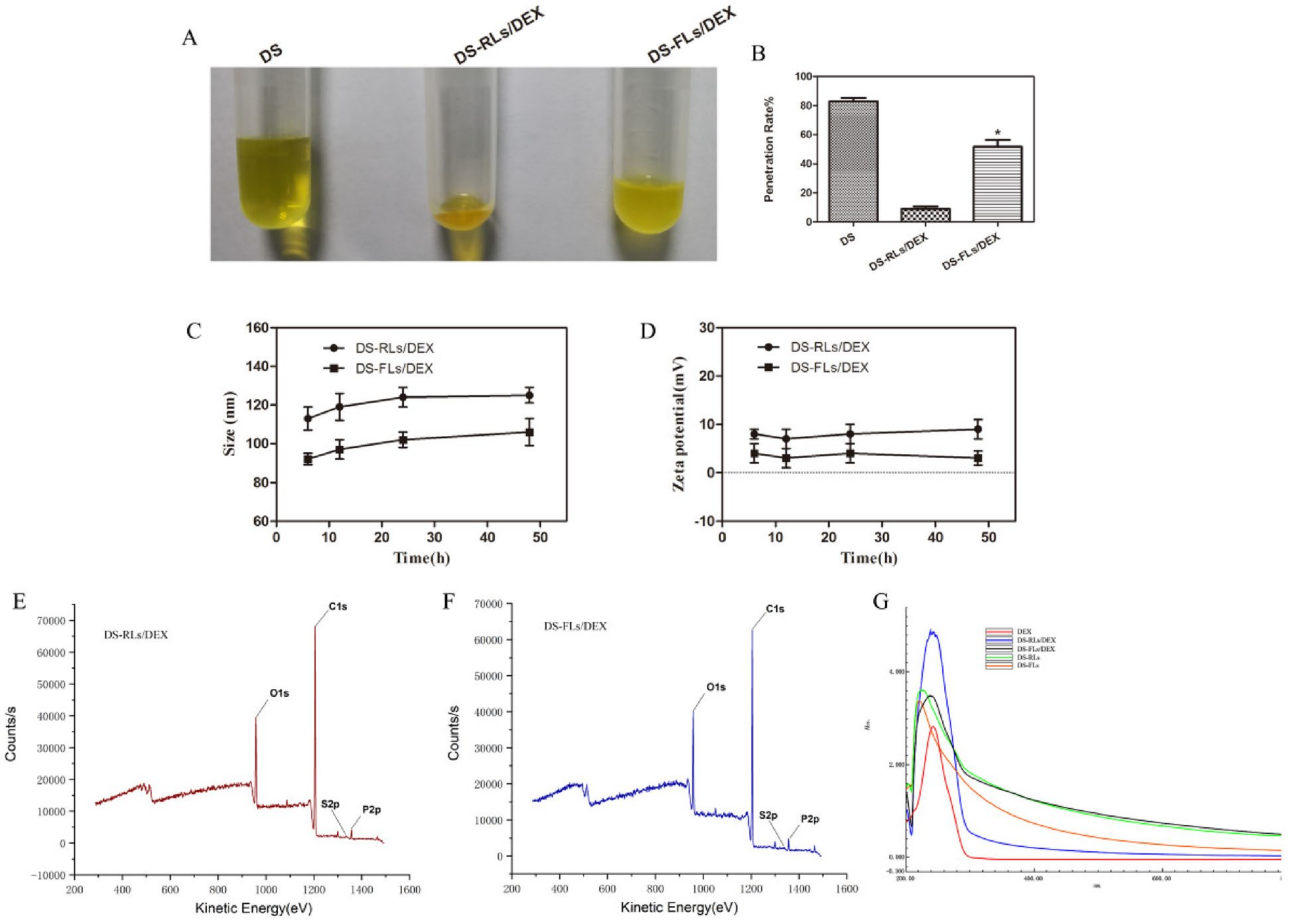
Deformability and stability of flexible liposome. (A) Typical images of C6 modified liposomes after extrusion method in tubes. (B) The penetration rate under 0.5 MPa pressure for 15 mins, *p < .05 vs DS-RLs/DEX. The size (C) and zeta potential (D) change of DS-RLs/DEX and DS-FLs/DEX in different time. XPS spectrum of DS-RLs/DEX (E) and DS-FLs/DEX (F). Full wavelength scanning spectrum of DS-RLs/DEX and DS-FLs/DEX by ultraviolet spectrophotometer.

Deformability of flexible liposomes membrane is a key factor for the permeation enhancing effect of lipid-based vehicles (Cao et al., 2017; Qindeel et al., 2020). As shown in Figure 3(A,B), the penetration rate of DS-FLs/DEX was 5.4 times higher than that of DS-RLs/DEX under the same pressure of 0.5 MPa, which showed the better deformability of DS-FLs/DEX. It is mainly because the edge activators NaDC that can be intercalated between the lipid bilayer, leading to a decline phase transition temperature of lipids and growth fluidity (Liu et al., 2021). The results indicated that, with the same size, the DS-FLs/DEX could be the more suitable carrier for pass through the smaller skin pore and has better transdermal permeability, which was beneficial to transdermal delivery. The stability of DS-RLs/DEX and DS-FLs/DEX were evaluated for 48 h, the change of size and zeta were shown in Figure 3(C,D). Both DS-RLs/DEX and DS-FLs/DEX were still homogeneous visually and there was no significant in two stability parameters at different time. The favorable stability of the DS-RLs/DEX and DS-FLs/DEX will exhibiting a strong resistance to breakage triggered in the complicated transdermal environment. The surface elements of DS-RLs/DEX and DS-FLs/DEX were analyzed by XPS. As showed in Figure 3(E,F), they can be observed sulfur elements on both DS-RLs/DEX and DS-FLs/DEX. In addition, sulfur elements are only present in the DS molecule. Therefore, it can be indicated that the DS successfully localized on the surface of DS-RLs/DEX and DS-FLs/DEX. The full wavelength scanning method of ultraviolet spectrophotometer was used to verify whether DEX was successfully encapsulated in liposomes. As showed in Figure 3(G), compared with the blank liposomes, the characteristic absorption peak of DEX can be obviously scanned in the drug-loaded liposomes. The results further confirmed that DEX was successfully encapsulated in DS-RLs/DEX and DS-FLs/DEX.

### 3.3. Biocompatibility

**Figure 4.**
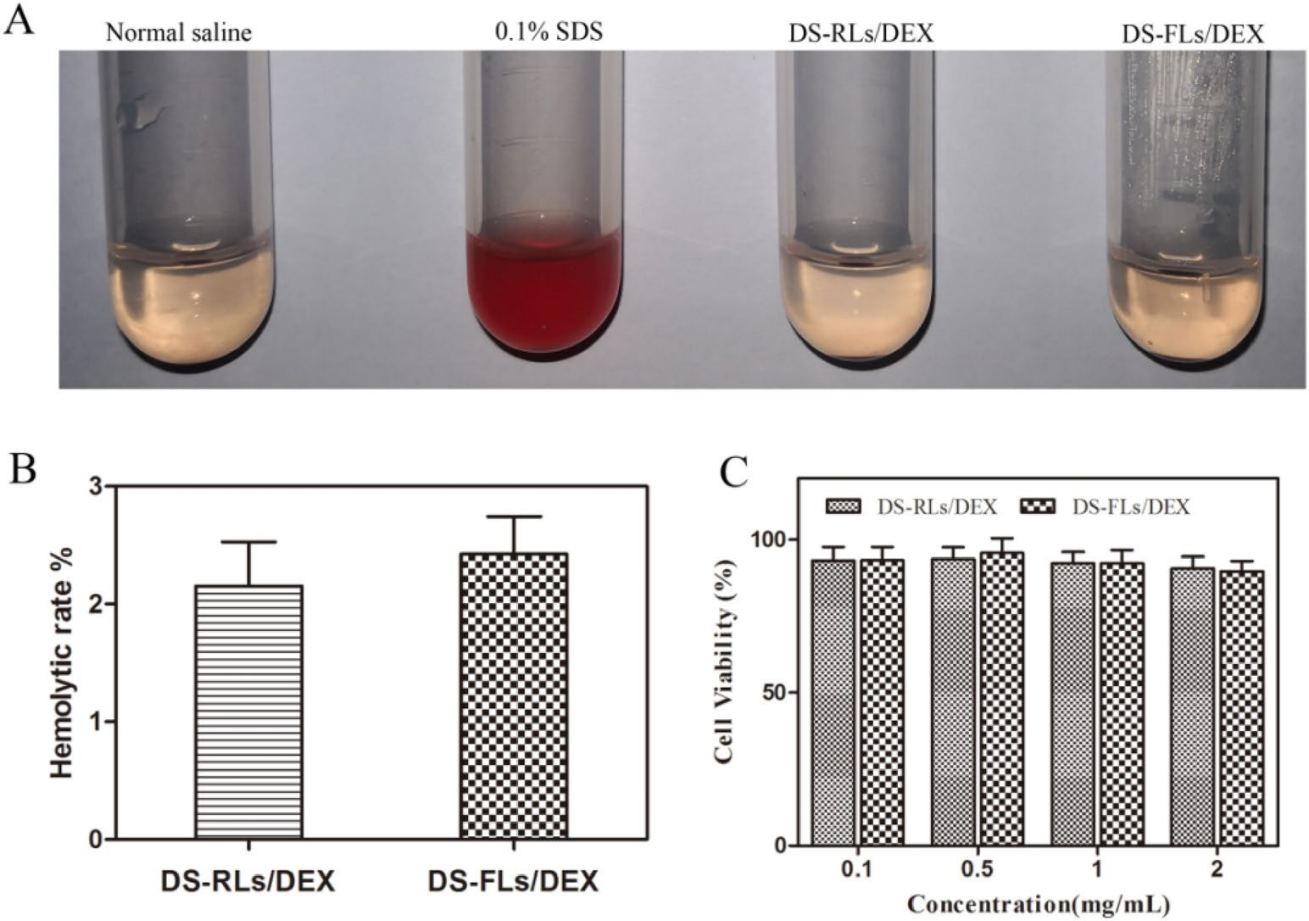
Biosecurity of DS-RLs/DEX and DS-FLs/DEX. (A) Hemolysis images and (B) statistics result of hemolysis ratio. (C) MTT assay to detect the cellular toxicity on HUVEC cells.

The hemolytic assay was conducted to investigate the safety of DS-RLs/DEX and DS-FLs/DEX. As shown in Figure 4(A,B), saline as negative control and 0.1% SDS as positive control, both DS-RLs/DEX and DS-FLs/DEX did not produce any remarkable hemolysis (<3%), which might be due to the anionic character of DS, which can shield the cationic character of DOTAP, thereby exhibiting less hemolytic toxicity. In vitro cytotoxicity of DS-RLs/DEX and DS-FLs/DEX was further evaluated by MTT assay. It was observed that DS-RLs/DEX and DS-FLs/DEX had little cytotoxicity to HUVECs cells at tested concentration of 0.1–2 mg/mL, as the results showed that all the cell viabilities were all over 90% (Figure 4(C)). These results revealed that both DS-RLs/DEX and DS-FLs/DEX have good biocompatibility and can be safely used for further research.

### 3.4. In vitro release studies

**Figure 5.**
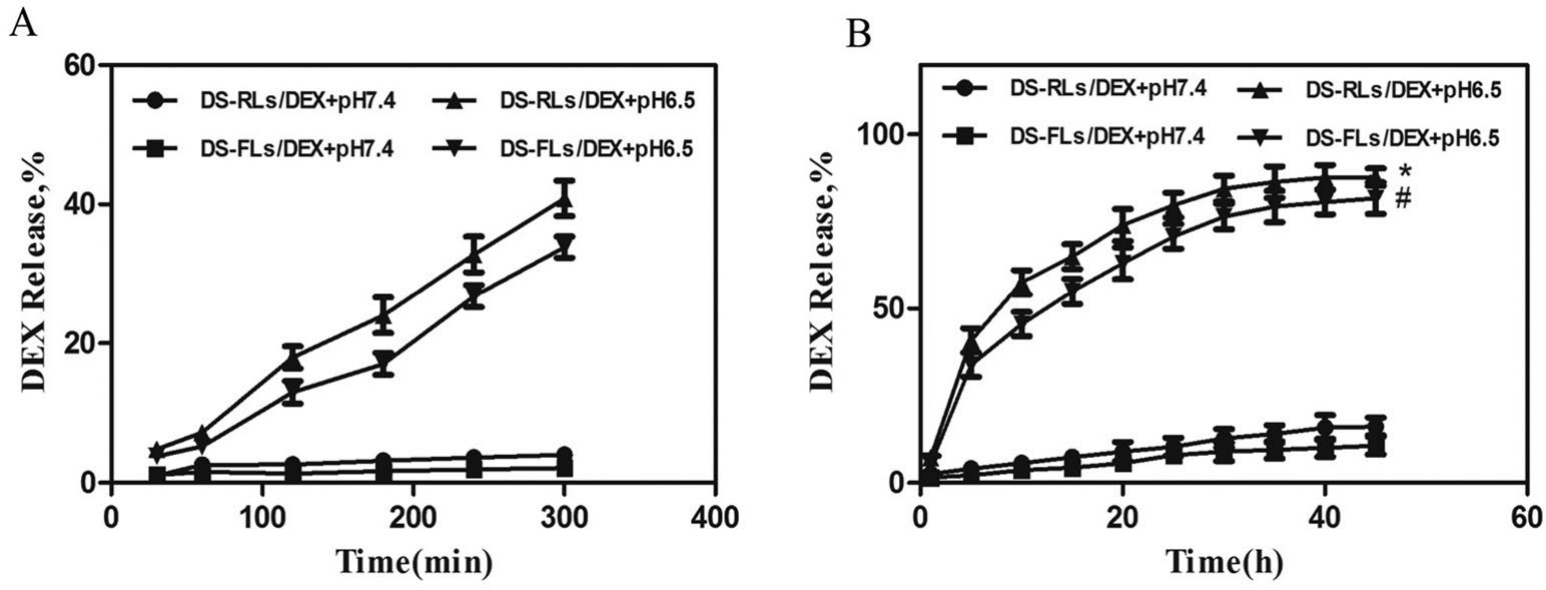
The DEX release of DS-RLs/DEX and DS-FLs/DEX in pH = 7.4 and 6.5 PBS for different times. (A) Burst release within 5 h. (B) Sustained release after 5 h. *p < .05 vs DS-RLs/DEX pH = 7.4, #*p* < .05 vs DS-FLs/DEX pH = 7.4.

In vitro release studies were performed at physiological pH (7.4) and inflammatory pH (6.5) to investigate the release pattern of DEX (Zhao et al., 2018). During the physiological pH environment, DEX release with almost the same pattern in both DS-RLs/DEX and DS-FLs/DEX groups. Only nearly 20% of the DEX was released within 48 h. However, in inflammatory pH solution, DS-RLs/DEX and DS-FLs/DEX exhibited an initial burst release of 40% and 33% within 5 h, respectively (Figure 5(A)). After initial burst release, DS-RLs/DEX and DS-FLs/DEX showed a sustained release and almost 90% of DEX was released at 48 h (Figure 5(B)). The initial burst release in inflammatory pH solution might be related to DEX present at the liposomes surface (Coburn et al., 2017; He et al., 2017). In addition, both DS-RLs/DEX and DS-FLs/DEX displayed significant pH-dependent drug release properties. The possible reason for this phenomenon is that DOTAP, the main material of liposomes, has better solubility under acidic conditions, which makes the two liposomes exhibit acid-sensitive drug release characteristics (Grace et al., 2021). The cumulative low drug release at physiological pH helps to reduce the systemic toxic side effects of DEX. Meanwhile, the cumulative highly drug release at pH6.5 was of great value specifically at inflammatory sites and would exert much better therapeutic effects in RA.

### 3.5. Targeting and anti-inflammatory in activated macrophages

**Figure 6.**
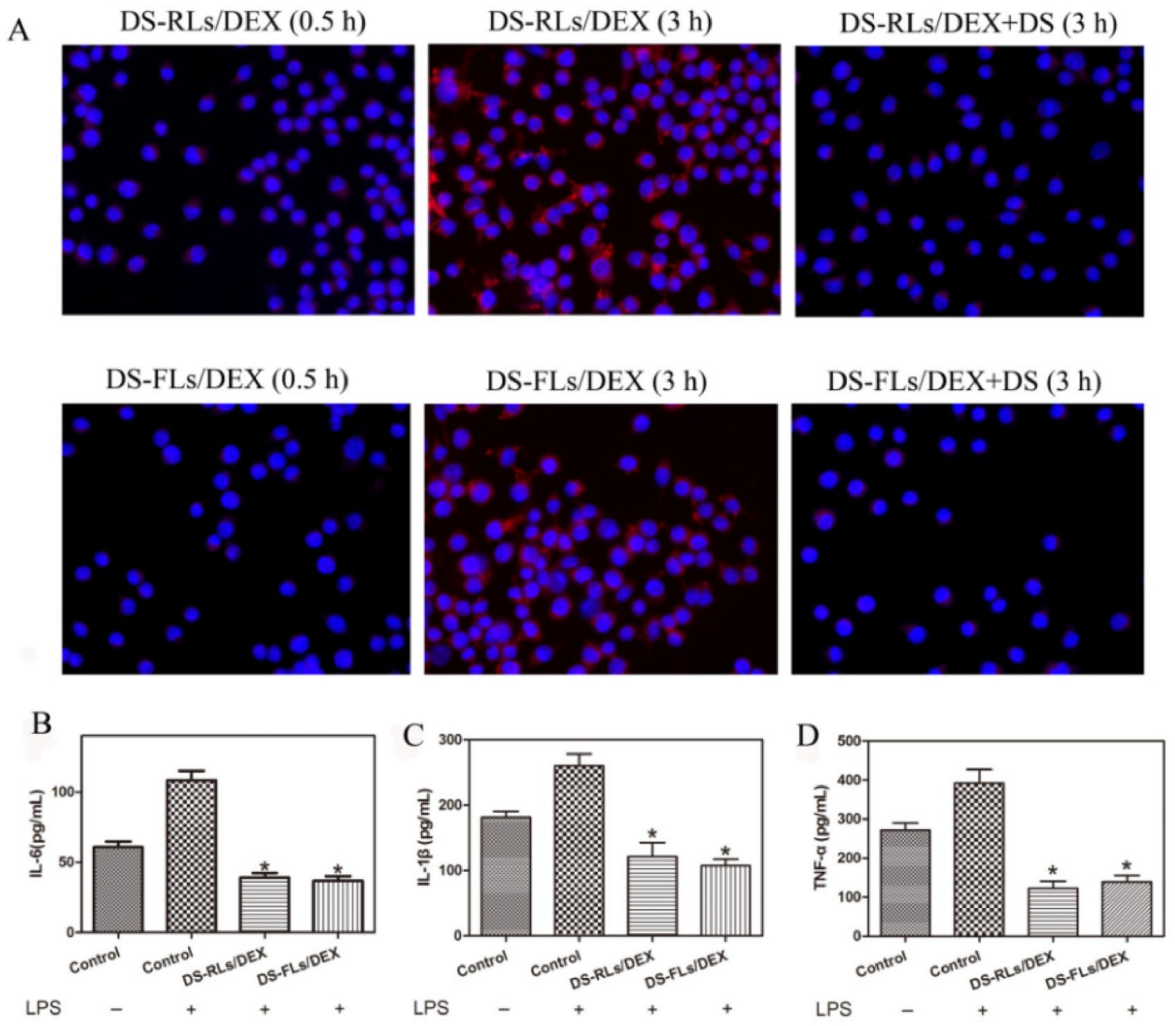
Cellular uptake and suppress the inflammation of DS-RLs/DEX and DS-FLs/DEX. (A) In vitro RAW264.7 cellular uptake of DIR-labeled liposomes after induced with LPS (100 ng/mL) by 0.5 and 3 h under fluorescence microscope (Scale bars = 200 nm). (B–D) The expression levels of IL-6, IL-1β and TNF-α after DS-RLs/DEX and DS-FLs/DEX incubation under LPS-activated RAW264.7 cells, *p < .05 vs control.

The targeting ability of the liposomes labeled with DIR toward the LPS activated RAW264.7 cells was observed under fluorescence microscope (Figure 6(A)). In the activated RAW264.7 cells, a few liposomes were found to accumulate around the cells at 0.5 h and a large amount of DIR-DS-RLs/DEX and DIR-DS-FLs/DEX were uptake, as observed through strong red fluorescence in 3 h. Nevertheless, in the blocking experiment, the fluorescence was weakened from the free DS addition in both DIR-DS-RLs/DEX and DIR-DS-FLs/DEX. These results confirmed the targeting ability of DS to activated RAW264.7 cells. To validate that the anti-inflammatory activity of DS-RLs/DEX and DS-FLs/DEX, the pro-inflammatory factor IL-6, IL-1β, and TNF-α in LPS-activated RAW264.7 cells were detected by ELISA kit. It has been reported that interleukin family and TNF-α play vital role in the RA progress and the expression of TNF-α could be suppressed by DEX (Wang et al., 2016). As shown in Figure 6(B–D), DS-RLs/DEX and DS-FLs/DEX remarkably inhibited the expression of IL-6, IL-1β, and TNF-α in LPS-activated RAW264.7 cells. These results implied that DS-RLs/DEX and DS-FLs/DEX showed better anti-inflammatory efficacy, which might be a promising candidate for RA therapy.

### 3.6. Characterization of DS-RLs/DEX hydrogel and DS-FLs/DEX hydrogel

Carbomer 934 is widely used in the preparation of hydrogels due to its good biocompatibility, nonirritant properties, and ability to form an immaculate hydrogel (Fu et al., 2002). In physical appearance, the two prepared DS-RLs/DEX hydrogel and DS-FLs/DEX hydrogel had homogeneous and transparent color. The particle size, PDI, zeta potential, and encapsulation efficiency of the liposomes were remained unchanged after embedding into hydrogels (data not shown). Other characterization of DS-RLs/DEX hydrogel and DS-FLs/DEX hydrogel were shown in Table 2, the pH values were found to be 6.03 ± 0.031 and 6.05 ± 0.028, respectively. Percentage drug content was 28.37% ± 1.21 and 28.16% ± 1.35, respectively. Spreadability values were found to be 6.09 ± 0.13, and 6.12 ± 0.26 cm/s, respectively. The retention time were found to be 6.69 ± 1.25 and 6.81 ± 1.47 h, respectively. These results implied that the DS-RLs/DEX hydrogel and DS-FLs/DEX hydrogel have the potential to be transdermally administered onto the skin.

**Table 2.** The characterization of DS-RLs/DEX hydrogel and DS-FLs/DEX hydrogel.

	pH value	Percentage drug content (%)	Spreadability values (m/s)	Retention time (h)
DS-RLs/DEX hydrogel	122 ± 18	+6.65 ± 1.5	0.215 ± 0.09	9.5 ± 1.9
DS-FLs/DEX hydrogel	112 ± 13	+2.55 ± 1.3	0.196 ± 0.6	10.1 ± 1.6

### 3.7. Ex vivo entrapment and permeation studies

**Figure 7.**
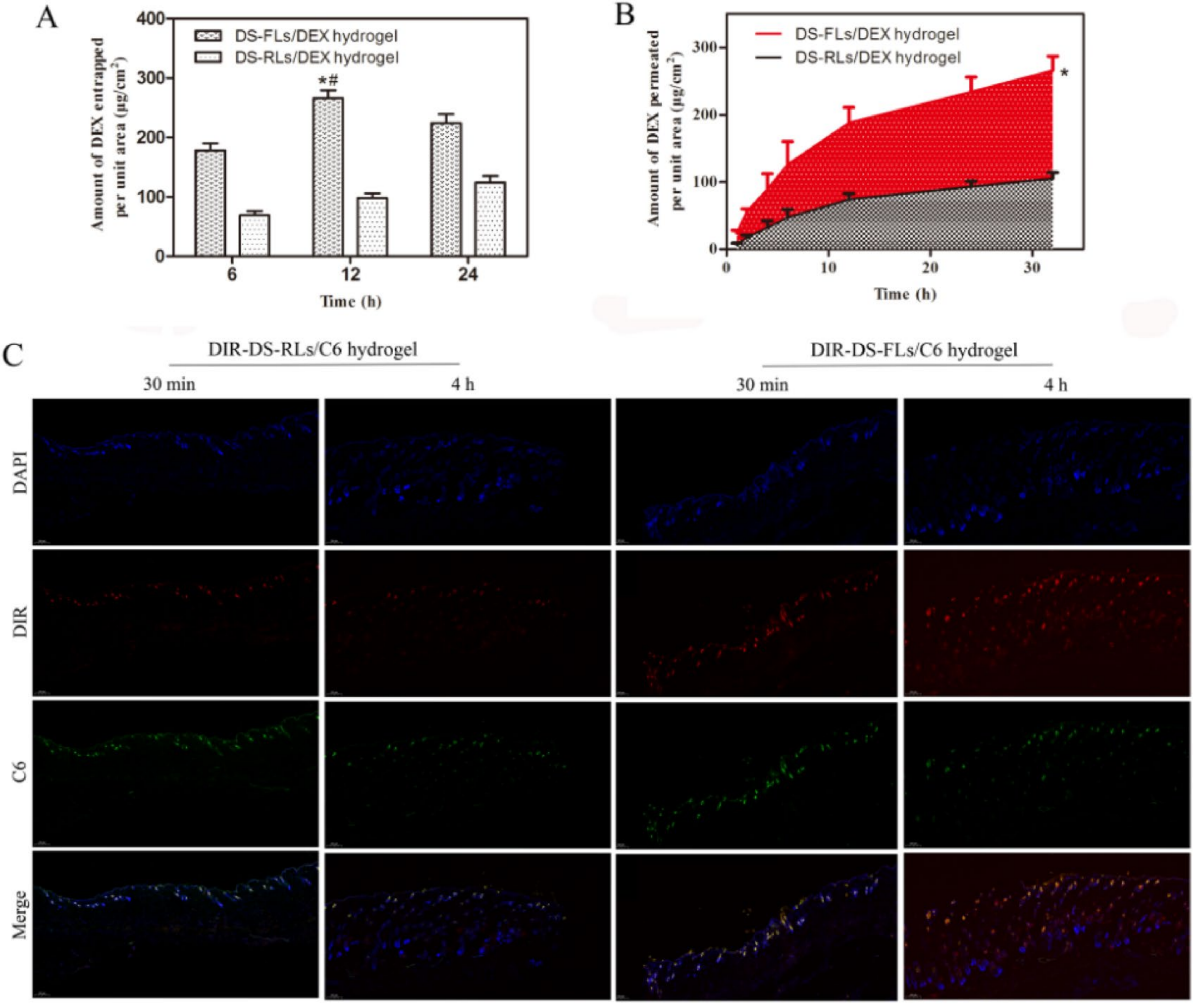
Ex vivo entrapment and permeation studies. (A) Amount of DEX entrapped per unit area from DS-RLs/DEX hydrogel and DS-FLs/DEX hydrogel. (B) Amount of DEX permeated per unit area from DS-RLs/DEX and DS-FLs/DEX hydrogel, *p < .05 vs DS-RLs/DEX. (C) Fluorescence images of skin treated with DIR-labeled DS-RLs/C6 hydrogel and DIR-labeled DS-FLs/C6 hydrogel at 30 min and 4 h (Scale bars = 200 nm).

Ex vivo entrapment and permeation studies were performed to investigate the capable of the vehicle to cross the major barrier, stratum corneum, for transdermal drug delivery. As showed in Figure 7(A), the amount of DEX entrapped in rats’ skin was determined in different time. For the DS-FLs/DEX hydrogel group, the DEX entrapment revealed at least 2-fold higher than DS-RLs/DEX hydrogel at each check point. The permeation studies showed that the DS-FLs/DEX hydrogel exhibited significantly higher DEX across the skin compared to DS-RLs/DEX hydrogel at each check point (Figure 7(B)). For the purpose of visualization evaluation, fluorescence-based liposomes penetration was observed using fluorescence microscope within different time intervals. As shown in Figure 7(C), the results showed that DIR-DS-FLs/C6 hydrogel had stronger DIR and C6 fluorescence intensity on the surface of active epidermis after 30 min transdermal administration. After 4 h of administration, the fluorescence intensity of the whole skin continued to increase, and it could effectively penetrate into the deep dermis. In addition, the fluorescence intensity of DIR-DS-FLs/C6 hydrogel group in hair follicles, sebaceous glands and sweat ducts was much stronger than that of DIR-DS-RLs/C6 hydrogel group, which was due to the better permeability of flexible liposomes. On the contrary, in the DIR-DS-RLs/C6 hydrogel group, the fluorescence intensity was weaker in viable epidermis, dermis and hair follicles. These results further confirmed that the deformability of liposomes can effectively enhance the transdermal efficiency of the drugs.

### 3.8. In vivo skin accumulation studies

**Figure 8.**
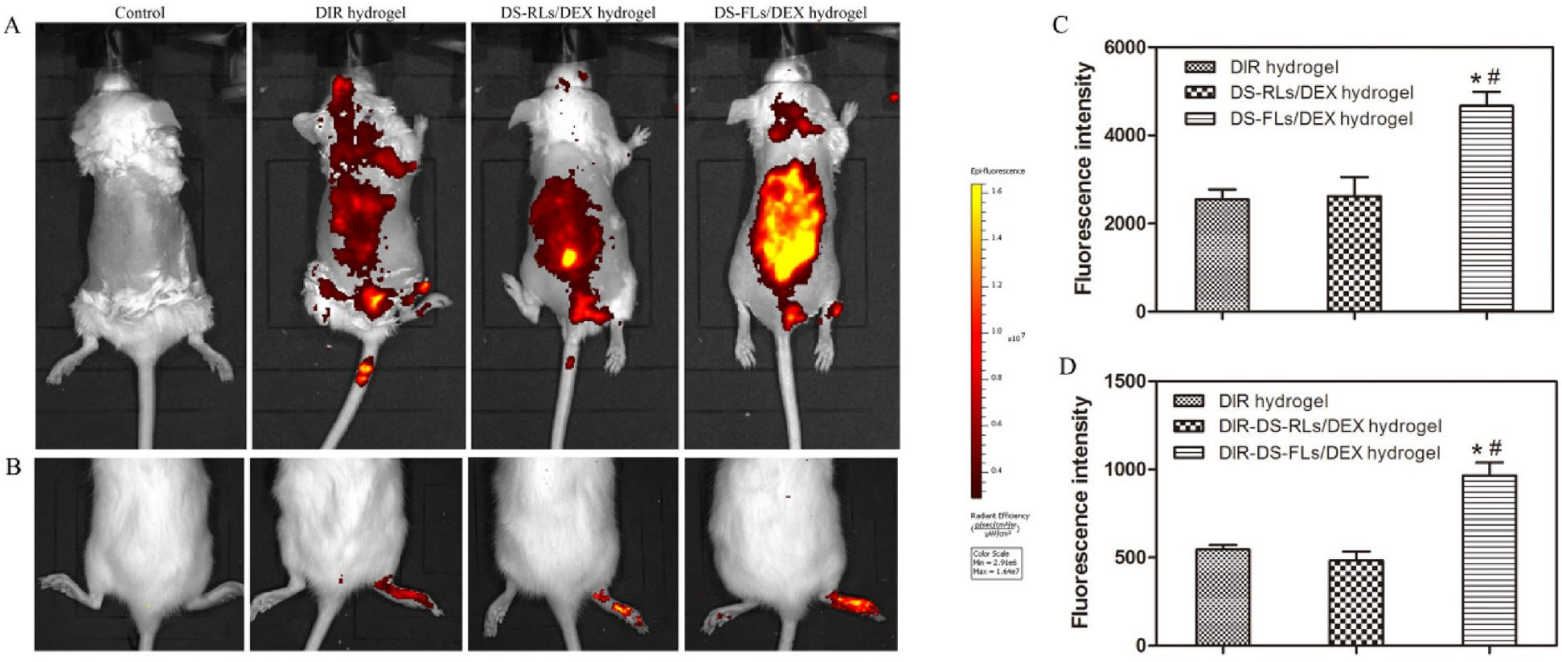
In vivo skin accumulation studies. (A) Skin accumulation of DIR-DS-RLs/DEX hydrogel and DIR-DS-FLs/DEX hydrogel detected by living image system. (B) The accumulation in joints of DIR-DS-RLs/DEX hydrogel and DIR-DS-FLs/DEX hydrogel detected by living image system. (C and D) Quantitative analysis of in-Vivo Images. *p < .05 vs DIR hydrogel, #*p* < .05 vs DIR-DS-RLs/DEX hydrogel.

To confirm the higher in vivo skin accumulation of the DS-RLS/DEX hydrogel, penetration analysis was performed by an in vivo imaging system (Figure 8(A)). There was a non-obvious fluorescence signal was found in the control group. Compared with free DIR hydrogel and DIR-DS-RLs/DEX hydrogel, the rats treated with DIR-DS-FLs/DEX hydrogel showed the maximum fluorescence, which further confirmed that DIR-DS-FLs/DEX hydrogel had higher skin permeability. In order to further verify the accumulation in vivo, quantitative analysis was performed (Figure 8(C)). After 4 h of administration, the fluorescence intensity of the rats in DIR-DS-FLs/DEX hydrogel treatment group was 4665 ± 324; the fluorescence intensity in DIR-DS-RLs/DEX hydrogel treatment group was 2623 ± 431; the fluorescence intensity of rats in DIR hydrogel treatment group was 2545 ± 226. The fluorescence intensity of DIR-DS-FLs/DEX hydrogel treatment group was about 1.7 times that in DIR-DS-RLs/DEX hydrogel treatment group and about 1.8 times that in DIR hydrogel treatment group.

To further investigate the accumulation properties of DS-RLs/DEX hydrogel at the RA joint site, the AIA rats were trans-dermally administered DIR-DS-RLs/DEX hydrogel, and performed by an in vivo imaging system (Figure 8(C,D)). Compared with free DIR hydrogel and DIR-DS-RLs/DEX hydrogel, the feet of the AIA rats treated with DIR-DS-FLs/DEX hydrogel showed the maximum fluorescence, which further confirmed that DIR-DS-FLs/DEX hydrogel can be effectively permeated and accumulated in the lesion site. The above results showed that DS-FLs/DEX hydrogel can significantly improve the skin permeability of DEX, and the delivery system is expected to be effective in the treatment of RA.

### 3.9. Pharmacokinetic studies

**Figure 9.**
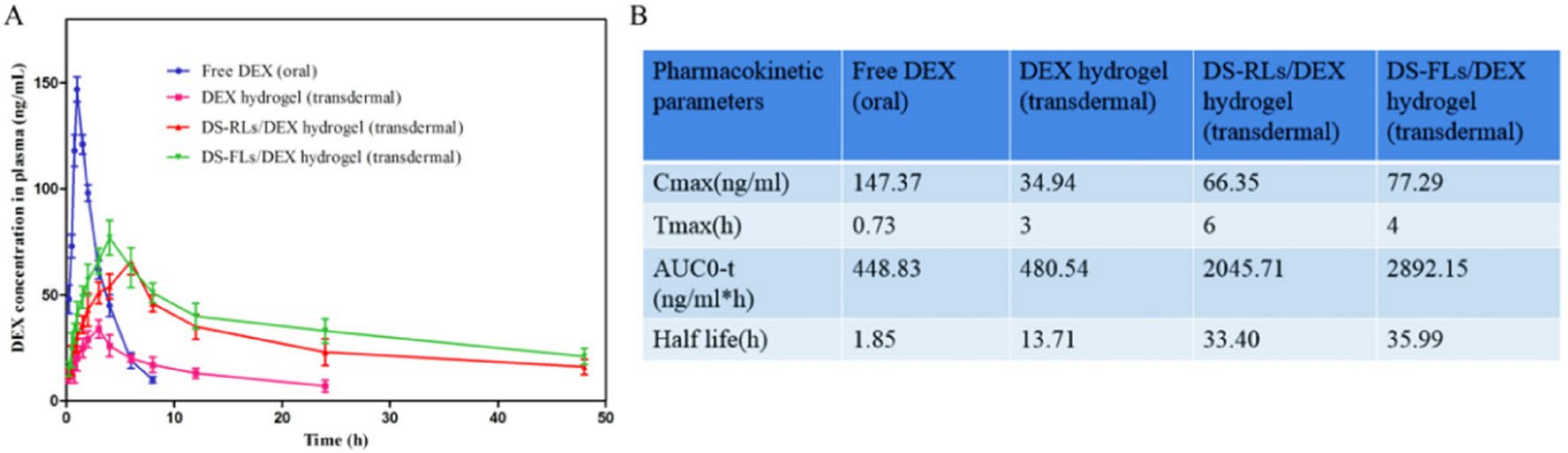
Pharmacokinetics study of DS-RLs/DEX hydrogel and DS-FLs/DEX hydrogel in AIA rats. (A) Plasma concentration-vs.-time curves. (B) Estimated pharmacokinetic parameters by using DAS 2.0 software.

The short plasma half-life of free DEX has severely limited its efficacy in RA therapy, and studies have shown that delivery systems can prolong the half-life of drugs and increase the therapeutic effect (Fu et al., 2002). In this study, the plasma half-life of DS-FLs/DEX hydrogel was evaluated by using the pharmacokinetics method. The plasma concentration-time distribution curves of the free DEX (oral), DEX hydrogel (transdermal), DS-RLs/DEX hydrogel (transdermal), and DS-FLs/DEX hydrogel (transdermal) are shown in Figure 9(A). After 10 h of administration, a little amount of the DEX was detected in the blood plasma neither by oral route (free DEX) nor transdermal route (DEX hydrogel), whereas a significantly higher amount of DEX was detected in DS-RLs/DEX hydrogel and DS-FLs/DEX hydrogel group. Plasma half-life of DS-FLs/DEX hydrogel was also found to be higher than DS-RLs/DEX hydrogel. AUC results exhibited that the AUC0-t of DS-FLs/DEX hydrogel group was much higher than the DS-RLs/DEX hydrogel. These results ensured that the DS-FLs/DEX hydrogel has the ability to prolong the plasma half-life and has the potential to improve the bioavailability of the DEX. In addition, the other pharmacokinetic parameters of the free DEX (oral), DEX-based hydrogels, DS-RLs/DEX hydrogel, and DS-FLs/DEX hydrogel are shown in Figure 9(B).

### 3.10. In vivo therapeutic effects

**Figure 10.**
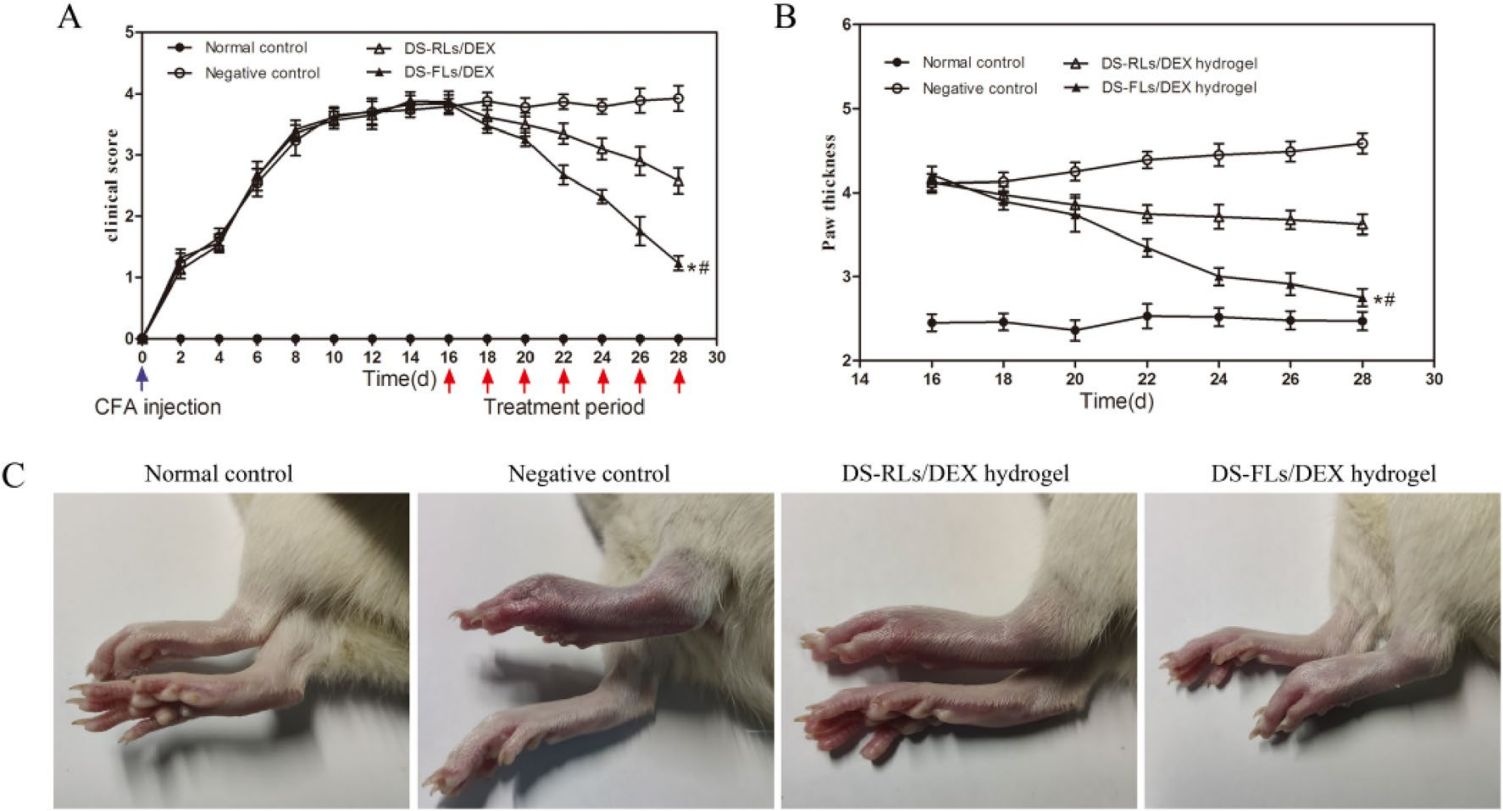
Therapeutic effects of DS-RLs/DEX hydrogel and DS-FLs/DEX hydrogel in AIA rats. The clinical score (A) and mean paw thickness (B), *p < .05 vs negative control, #*p* < .05 vs DS-RLs/DEX hydrogel. (C) Image of the hind legs from macroscopic observation.

To evaluate the anti-inflammatory effects, the DS-FLs/DEX hydrogel and DS-RLs/DEX hydrogel administered AIA rats by transdermal delivery. Subsequently, the photographs of hind paws, clinical score and paw thickness for the rats were measured every other day for 28 days after the first immunization. As shown in Figure 10(A), compared with negative control group, rats treated with DS-FLs/DEX hydrogel and DS-RLs/DEX hydrogel possessed obviously lower clinical score. Of special interest is the DS-FLs/DEX hydrogel treated group displayed the lowest clinical score after seven times of transdermal administration. Furthermore, the paw thickness was used as a direct indicator extent of paw swelling in the AIA. As shown in Figure 10(B), both of DS-FLs/DEX hydrogel and DS-RLs/DEX hydrogel showed prominent reduction of paw thickness of the AIA rats. Notably, the DS-FLs/DEX hydrogel treatment group had a more pronounced decrease in paw thickness than the DS-RLs/DEX hydrogel treatment group, which was consistent with the results of clinical score. More importantly, after the treatment, there was no difference in the paw thickness between the DS-FLs/DEX hydrogel treatment group and the normal control group. In addition, similar results were obtained based on the macroscopic observation of hind legs morphology (Figure 10(C)). These results suggested that the therapeutic effect of DS-FLs/DEX hydrogel was superior to that of DS-RLs/DEX hydrogel, possibly due to the higher deformability and permeability of DS-FLs/DEX to arthritic skin.

**Figure 11.**
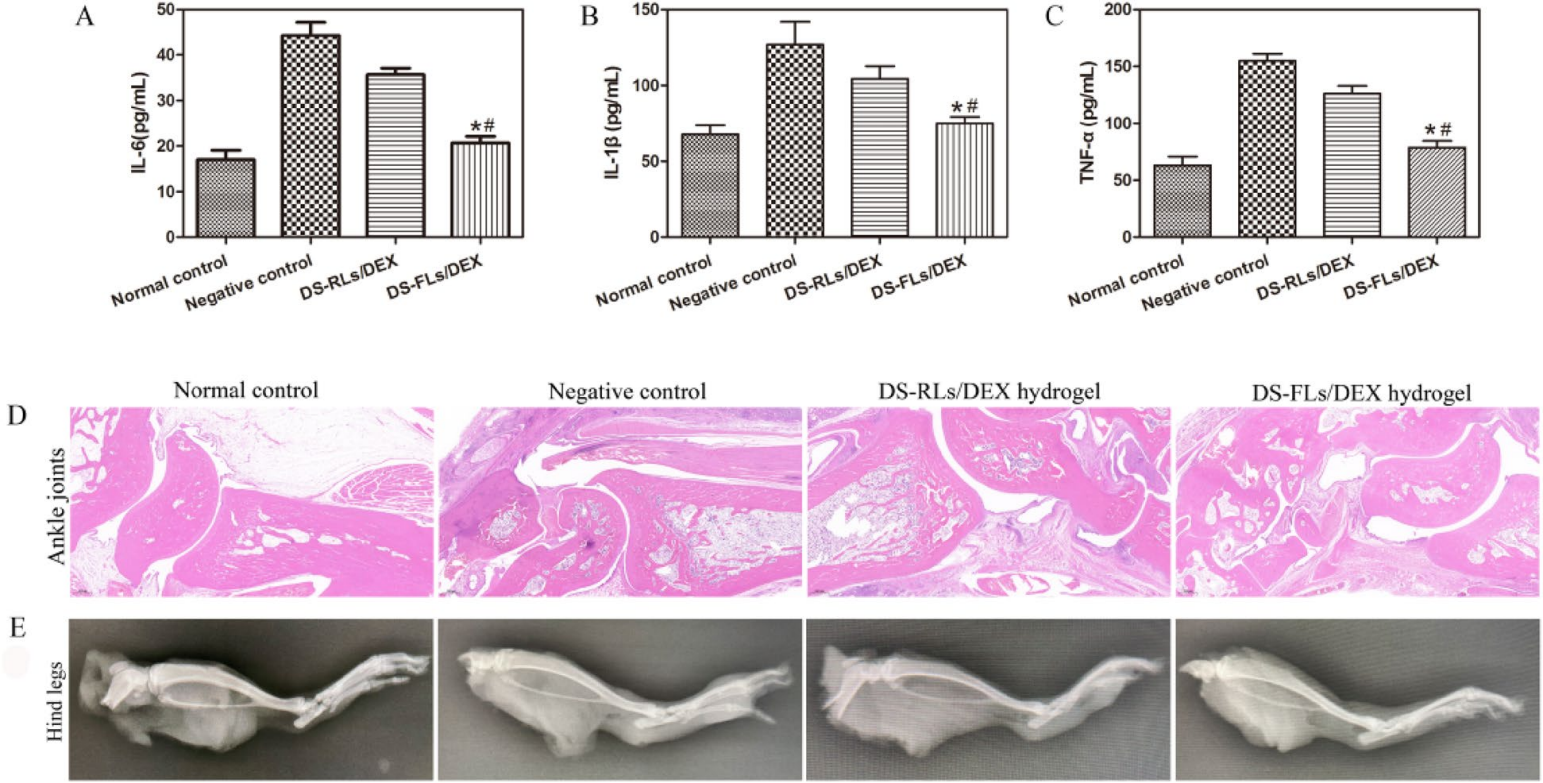
In vivo proinflammatory cytokines assay and Histological analysis of AIA rats after treated with DS-RLs/DEX hydrogel and DS-FLs/DEX hydrogel. (A–C) The expression levels of IL-6, IL-1β and TNF-α in joint tissue, *p < .05 vs negative control, #*p* < .05 vs DS-RLs/DEX hydrogel. HE staining of ankle joints (D) (Scale bars = 200 nm). (E) X-ray images of hind paws.

The pro-inflammatory cytokines TNF-α, IL-1β, and IL-6 play a very important role in the occurrence and development of RA. DEX has a strong anti-inflammatory effect and can reduce the expression of inflammatory factors (Wang et al., 2020). As shown in Figure 11(A–C), Both DS-RLs/DEX hydrogel and DS-FLs/DEX hydrogel treated groups reduced the expression levels of TNF-α, IL-1β and IL-6 to some extent in compared with the negative control group. Moreover, DS-FLs/DEX hydrogel showed lower inflammatory cytokines level than DS-RLs/DEX hydrogel group. The histopathological results of the ankle joint showed that the negative control group had severe ankle joint hyperplasia with high inflammatory cell infiltration and cartilage damage, and it was difficult to identify the complete joint cavity. In contrast, DS-RLs/DEX hydrogel and DS-FLs/DEX hydrogel treated groups significantly reduced synovial inflammation and cartilage erosion in AIA rats. Notably, the cartilage of AIA rats in the DS-FLs/DEX hydrogel treated group was normal, with a clear interface and less inflammatory cell infiltration (Figure 11(D)). The X-ray assessed bone erosion in AIA rats (Figure 11(E)). Compared with the DS-RLs/DEX hydrogel treated group, the DS-FLs/DEX hydrogel treated group could alleviate the destructive effect of inflammation on the bone parenchyma, and there was no significant difference compared with the normal control group. These results indicated that DS-FLS/DEX hydrogel can enhance the accumulation of DEX in the inflammatory joints, further improve the anti-inflammatory effect and relieve the symptoms of paw swelling, which is expected to be a candidate nanocarrier for the clinical treatment of RA.

### 3.11. In vivo evaluation of biocompatibility

**Figure 12.**
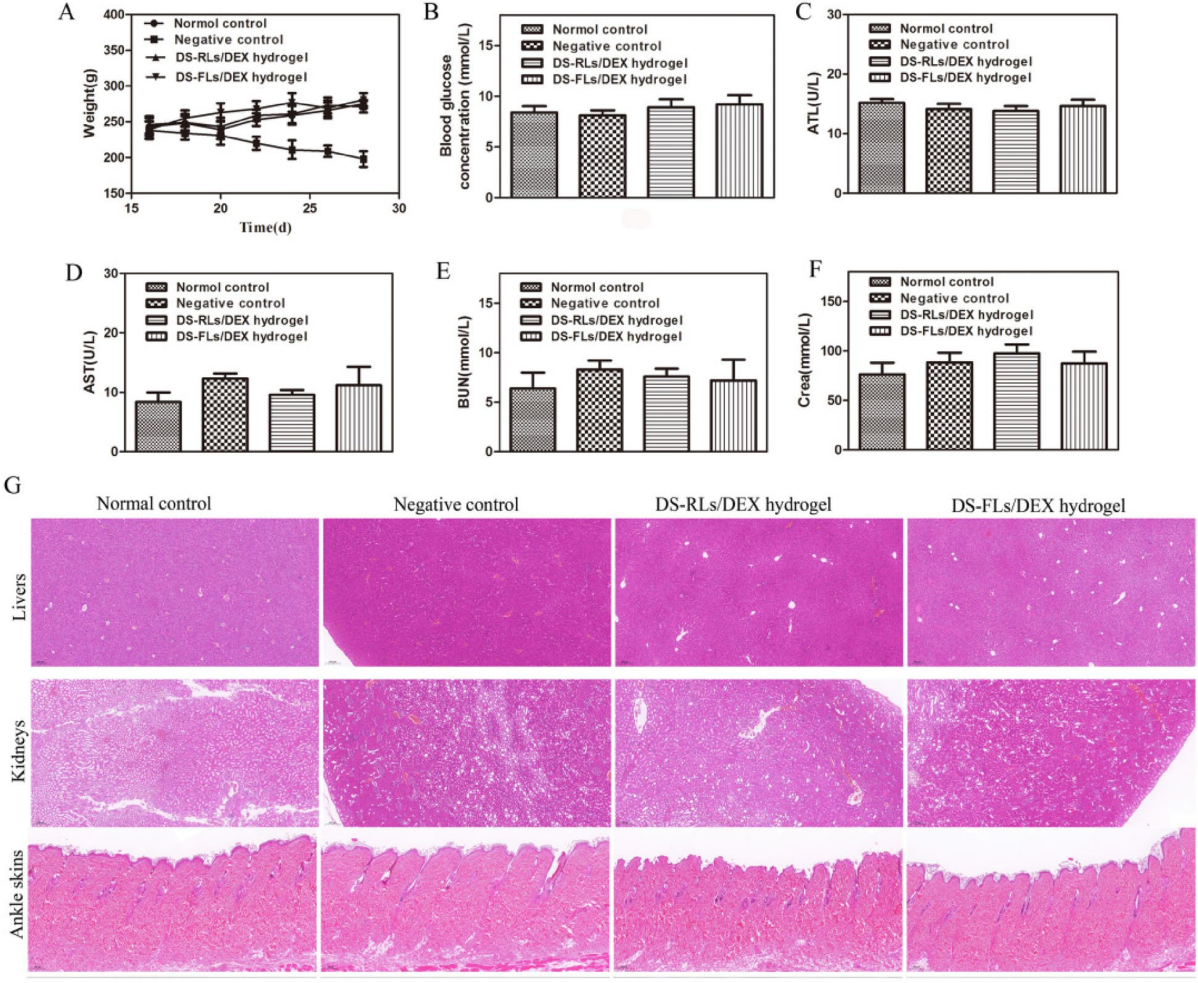
Evaluation of biocompatibility of DS-RLs/DEX hydrogel and DS-FLs/DEX hydrogel in vivo. (A) Body weight. (B) Blood glucose concentration after 15 days of administration. (C) ALT, (D) AST, (E) BUN, and (F) Crea levels in serum. (G) HE staining histological sections of liver, kidney and ankle skin after DS-RLs/DEX hydrogel and DS-FLs/DEX hydrogel treatment (Scale bars = 200 nm).

The body weight change is a regular indicator of systemic toxicity, and the body weight of AIA rats was monitored during the treatment. In the whole treatment process, all the rats in the administration group did not have anorexia, anxiety and other abnormal symptoms. Compared with the negative control group, the body weight of rats in each treatment group continued to gently increase (Figure 12(A)), indicating that our treatment will not lead to weight loss. In addition, as long-term use of DEX is often prone to side effects of hyperglycemia, the blood glucose level of rats was measured after 4 h administration on days 15. The average blood glucose of DS-RLs/DEX hydrogel and DS-FLs/DEX hydrogel both were within the normal range, indicating that our therapeutic will not lead to blood glucose disorder (Figure 12(B)). The various parameters were also detected, and special attention was paid to the liver (such as ALP, AST) and kidney (such as CRE, BUN) (Figure 12(C–F)). Similarly, all indicators were confirmed to be within the normal range, indicating that there was no significant damage to these organs. HE further evaluated the staining of major organs of tissue injury (Figure 12(G)), and no obvious histopathological abnormalities or lesions were observed in each organ. Arthritic skin sections showed normal physiological structure and cell morphology. Based on these results, all these data indicate that DS-RLs/DEX hydrogel and DS-FLs/DEX hydrogel are nontoxic after transdermal administration.

## 4. Conclusions

In summary, an activated-macrophage targeted flexible liposome gel DS-FLs/DEX hydrogel, was prepared by filming-rehydration method and used as a transdermal formulation for effective RA treatment. The type of edge activators, mass ratio of DOTAP: NaDC, DOTAP: CHOL and DEX concentration were optimized through the particle size and encapsulation efficiency. Afterwards, the prepared DS-FLs/DEX had a uniformly distributed particle size of around 100 nm, homogeneous fusiform shape, good deformability, and commendable biocompatibility, which assured the prepared TDDS for the subsequent in vivo studies. In vitro, DS-FLs/DEX exhibited sustained acid-sensitive drug release characteristics and high uptake in LPS activated macrophages. In addition, DS-FLs/DEX endowed the encapsulated DEX with improved skin permeation ability. Furthermore, in vivo skin permeation and accumulation study showed DS-FLs/DEX hydrogel can significantly improve the skin permeability and accumulation of DEX. Animal model experiments exhibited a significant therapeutic efficacy of the DS-FLs/DEX hydrogel. Histopathological features and biochemical analysis further confirmed the reduction of inflammation signals. Main organs histopathological features exhibited that at therapeutic concentration, DS-RLs/DEX hydrogel produced no side effect as compared with normal control. This research demonstrated that DS-RLs/DEX hydrogel can be a great potential transdermal drug delivery system for RA therapy.
